# Racial disparities in youth pretrial detention: a retrospective cohort study grounded in critical race theory

**DOI:** 10.1186/s40352-022-00203-8

**Published:** 2023-03-08

**Authors:** Andy Wen, Noah R. Gubner, Michelle M. Garrison, Sarah Cusworth Walker

**Affiliations:** 1 University of Washington School of Medicine, Seattle, USA; 2 Department of Psychiatry, Yale School of Medicine, New Haven, USA; 3 University of Washington School of Public Health, Seattle, USA; 4 Department of Psychiatry and Behavioral Sciences, University of Washington School of Medicine, Seattle, USA; 5 Department of Public Health, Purdue University, West Lafayette, USA

**Keywords:** Juvenile justice, Juvenile detention, Pretrial detention, Critical race theory, Racial disparities, Incarceration

## Abstract

**Background and method:**

Pretrial detention makes up 75% of juvenile detention admissions and contributes to the disproportionate contact of minoritized youth in the juvenile carceral system. Given that prior evidence largely examines differences between Black and white youth, this study expands research on disproportionate contact in the pretrial detention setting to Hispanic/Latinx, Indigenous, and Asian youth. With a sample of over 44,000 juvenile cases in a northwest state, we used a generalized linear mixed model to estimate the effect of individual level characteristics while accounting for the random effect of differences at the county level. Additionally, we utilized Critical Race Theory (CRT) in formulating our theoretical model and predictions and apply CRT in our analysis and discussion of our results. In doing so we hope to build upon its application in public health discourse for naming and deconstructing processes that lead to unjust social and health stratification.

**Results:**

After factoring in gender, age, crime severity, previous offenses, and variation between counties, our analyses show that Black, Hispanic/Latinx, and American Indian/Alaskan Native youth are more likely to experience pretrial detention than white youth. The likelihood of pretrial detention for Asian youth and for youth identified as “Other” or “Unknown” was not significantly different from white youth.

**Conclusions:**

As the iatrogenic effects of detention are disproportionately imposed upon youth of color—particularly Black, Indigenous, and Hispanic/Latinx youth—the disparities present in our study reveal further evidence of institutional racism. In this way, we can see how this carceral process operates as a mechanism of racialized social stratification as put forth by CRT. Considering implications for policy or further research, persistent disparity highlights an enduring need for building or strengthening diversion programs and alternatives to the carceral system, with emphasis on those that are culturally responsive.

## Introduction and background

Youth of color are persistently overrepresented in the juvenile carceral system,^1^ most often referred to as “disproportionate minority contact” (Kempf-Leonard, 2007; Piquero, 2008). For example, while 53% of the juvenile population is white, non-Hispanic youth, they represent 33% of incarcerated youth, whereas Black youth made up 14% of the population and 40% of incarcerated youth (Rovner, 2014). As pretrial detention accounts for 75% of all admissions to detention facilities (Census of Juveniles, 2017), it is critical for researchers and policymakers to more fully understand how this experience impacts youth outcomes. Pivotally, detaining youth prior to adjudication has been shown to result in worse legal outcomes at later stages of processing. Examples include detained youth being more likely to have petitions filed for further proceedings, lower likelihood of petition dismissal, more severe sentences, and greater likelihood of the youth being removed from home (Feld, 1989; Leiber & Fox, 2005; Rodriguez, 2010). For youth with little prior contact with the carceral system, pretrial detention has been shown to be associated with increased recidivism (Walker & Herting, 2020). Additionally, the detention setting itself can expose confined youth to experiences presumed to present direct harm, including sexual abuse and harassment, physical violence, placement in restraints or solitary confinement, and further psychological trauma (Burrell, 2013). Furthermore, incarceration during adolescence has been shown to be independently associated with worse physical and mental health outcomes later in life (Barnert et al., 2017; Massoglia, 2008). Thus, racial inequities can result in disproportionate exposure to harm.

### Theoretical perspectives

Various theoretical perspectives are applied when considering the root causes of racial/ethnic disparities in juvenile pretrial detention. Among reviewed studies, only Secret and Johnson (1997), through their discussion of “conflict theory” explicitly name racism (along with classism) as a feature of the juvenile carceral system that underpins disparities. Moreover, Bishop and Frazier (1996) alone definitively state in their conclusion that they “see evidence of institutional racism,” while other studies prefer terms such as “bias” or “discrimination,” which can be seen as more neutral and often ascribe blame to individuals (e.g., a biased judge determining sentencing) rather than broad systems. This may be reflective of the general reluctance within health and criminal justice discourse to name structural racism as a root cause of inequities (Bailey et al., 2017).

Reluctance may also be due to the paucity of work that centers Critical Race Theory (CRT) and a Public Health Critical Race praxis (PHCR) (Delgado & Stefancic, 2001; Ford & Airhihenbuwa, 2010). Using this framework, we can be explicit that categories of race and ethnicity (as well as gender) in our (or any) study are social constructs and cannot represent any innate or biological characteristics of people. Racialization as a verb, rather than race as a noun, actively results in social stratification of populations according to historically entrenched, racist hierarchies defined by white supremacy (Ford & Airhihenbuwa, 2010; Müller-Wille, 2014). CRT further reveals the “ordinariness” of racism in that racism is ubiquitous and embedded in societal structure and institutions (Ford & Airhihenbuwa, 2010). The embeddedness and persistence over time and across place and institutions is the manifestation of what CRT calls structural determinism, which underlies theorization of structural racism as a fundamental cause of racial and ethnic health disparities (American Public Health Association, 2001; Bailey et al., 2017; Ford & Airhihenbuwa, 2010). Taken together, we can posit that the “justice system” is non-immune from institutional racism and is an effective tool for generating racialized social and health stratification through its disproportionate impacts on people of color (Massoglia, 2008). As such, disproportional incarceration has been used in literature as a measure of structural racism that predicts health outcomes (Lukachko et al., 2014; M. Wallace et al., 2017; M. E. Wallace et al., 2015). This is in alignment with Ruth Wilson Gilmore's (2007) definition of racism as “the state-sanctioned and/or extra-legal production and exploitation of group-differentiated vulnerabilities to premature death.” Considering racism as a fundamental cause of disparities may additionally explain observed stratified differences in other predictors of youth detention (discussed in greater detail below), such as income, single-parent households, or school attendance.

**Fig. 1:**
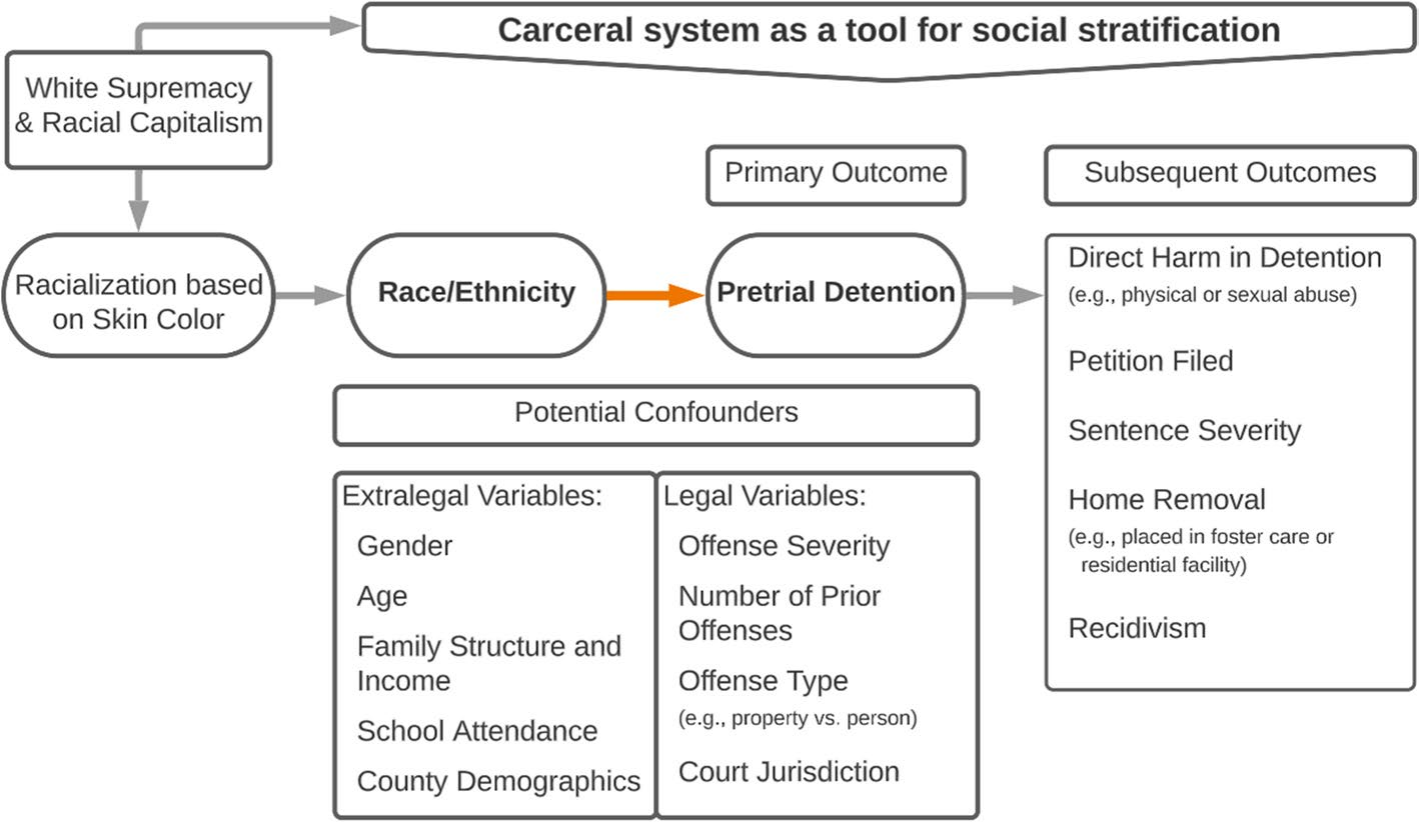
Theoretical model

Applying CRT to our study, we hold that the long historical record of racism and white supremacy informs our predictions and our theoretical model as displayed in Fig. 1. McDowell (2019) summarizes literature demonstrating how the post-emancipation social construction of “the Black Criminal” by law and social sciences underpins the carceral system as a tool for racial governance. Overt historic examples of racial governance via constructing the Black criminal include Jim Crow laws or differential policing based on crack or powder cocaine. Taken further, we may include construction of the “Indigenous Savage,” the “Latino Illegal Alien,” and what was once the “Yellow Peril” and now the “Model Minority” as potential predictors underlying racial disparities in the carceral system and beyond.

### Correlates of pretrial detention

In the currently available literature, several factors other than race have been shown to be predictors of pretrial detention. Legal predictors, or factors related to the offense or offense history, include variables such as prior offenses, severity of offense, and type of offense (e.g., personal or property) (Armstrong & Rodriguez, 2005; Bishop et al., 2010; Lowery & Smith, 2020; Rodriguez, 2010). Regarding social characteristics, a few studies have shown that measures of “concentrated disadvantage” or “structural disadvantage” predict pretrial detention (Lowery & Smith, 2020; Rodriguez, 2010). Conversely, “concentrated affluence” has been associated with a decreased likelihood of pretrial detention (Lowery & Smith, 2020). Specific social variables shown to predict pretrial detention include youth who were not attending school, youth with lower family incomes, and youth that lived in single-parent households (Armstrong & Rodriguez, 2005; Lowery & Smith, 2020; Rodriguez, 2010).

However, some studies have shown racially disparate impacts for youth in similar social contexts. For example, Leiber (2013) has shown that for Black youth in single-parent households, the risk of pretrial detention was 2.5 times higher than that of white youth in single-parent households. Leiber & Fox (2005) demonstrated an increased likelihood of release for white youth in single-parent households but decreased likelihood of release for Black youth in single-parent households. Drug use in white youth did not impact detention decisions whereas drug use increased likelihood of detention for Black youth (Leiber & Fox, 2005). These findings suggest race may be an independent predictor of pretrial detention.

To that end, numerous studies have shown increased risk of pretrial detention for Black youth (Armstrong & Rodriguez, 2005; Bishop & Frazier, 1996; Bortner & Reed, 1985; Leiber, 2013; Leiber & Fox, 2005; Lowery & Smith, 2020; Rodriguez, 2010; Secret & Johnson, 1997; Wu, 1997). To our knowledge, only two studies have shown a decreased likelihood of detention for Black youth, however the lower detention rates were posited to be a mechanism of “self-correction” as compensation in a system with arrest policies that disproportionately impact Black youth (Lau et al., 2018; Rodriguez, 2007). Both studies used samples from a single urban county and Lau et al. (2018) also examined only outcomes after first arrest in a population restricted to youth insured through Medicaid. For Hispanic/Latinx youth, Armstrong and Rodriguez (2005) and Rodriguez (2010) revealed an increased likelihood of pretrial detention, whereas Lau et al. (2018) and Lowery and Smith (2020) did not find a significant difference compared to white youth. With respect to American Indian youth, Rodriguez did not find a significant difference in her 2007 study, however, in her 2010 study with a much larger sample size spanning an entire state (*n* = 23,156 vs. *n* = 3060) there was an increased likelihood of detention for this population compared to white youth. Asian and Pacific Islander youth are predominantly omitted from these studies either without mention or included in the categorical “other.”

### Objective and hypothesis

With the majority of studies on juvenile pretrial detention being over a decade old, our study provides a needed update to the aging literature. The continued evaluation of disparities in pretrial detention is critical as it serves to audit a carceral system in a perpetual state of reform that emphasizes addressing disproportionate minority contact. Evidence of poor outcomes for youth who experience pretrial detention adds to the importance of this work. As prior studies have predominantly provided evidence on disparity within the dichotomy of Black and white youth, the present study is a significant contribution to racial and ethnic disparities specific to the pretrial detention setting. We present findings from one of the largest sample sizes to date and include adequate representation of Hispanic/Latinx, American Indian/Alaskan Native, as well as Asian youth. The inclusion of a large sample of Asian youth appears to be a unique contribution. Given the documented racial disparities in the juvenile carceral system at large and the embeddedness of structural and institutional racism, this study examines the following hypothesis: Youth of color—particularly Black, Hispanic/Latinx, and Indigenous youth—face a higher likelihood of pretrial detention compared to white youth.

Lastly, in addition to utilizing CRT in formulating our theoretical model and predictions, we apply CRT in our analysis and discussion of our results. In doing so we hope to build upon its application in public health discourse for naming and deconstructing processes that lead to unjust social and health stratification.

## Methods

### Sample and data sources

Data were obtained from an administrative dataset from 32 court jurisdictions and originally gathered between January 2002 and December 2015 for the purpose of evaluating a juvenile detention reduction initiative. The Juvenile Detention Alternatives Initiative (JDAI), as developed by the Annie E. Casey Foundation, was a multipart reform initiative intending to “reduce reliance on the use of detention for low-risk young people in the juvenile justice system prior to disposition” (Guckenburg et al., 2019). The initiative was funded and implemented in stages within 8 jurisdictions with the other 24 jurisdictions serving as controls. For each intervention jurisdiction and its three assigned control jurisdictions, juvenile filings spanning 5 years were collected—starting 2 years prior to the start of the initiative in the intervention county and continuing for 3 years after the initiative started. Cumulatively, the sample included youth with any filed offense in a juvenile court across 32 regional jurisdictions in a northwest state from January 2002 through December 2015, *n* = 46,124. For analysis, we excluded youth with missing data regarding pretrial detention (*n* = 1814), resulting in a final analytic sample of 44,310.

The sample was predominantly male (73%; 27% female) and race/ethnicity categories included Caucasian (67.3%), Black/African American (9.3%), Asian (2.6%), American Indian/Alaskan Native (3.7%), Hispanic/Latinx (16.0%), Native Hawaiian/Pacific Islander (0.03%), and Unknown/Other (1.2%). The mean age at first ever offense was 15.1 years. Full descriptive statistics are found in Table 1.

**Table 1:** Descriptive statistics

		Count	Column N %
	Total	44,310	100%
Race	Black / African American	4130	9%
	Asian	1134	3%
	American Indian / Alaskan Native	1626	4%
	Hispanic / Latinx	7091	16%
	White	29,801	67%
	Other / Unknown	528	1%
Gender	Female	11,956	27%
	Male	32,342	73%
	Other / Unspecified	12	0%
Age at Qualifying Offense	16+ Years Old	19,751	45%
	14–16 Years Old	16,685	38%
	<14 Years Old	7874	18%
Qualifying Offense Severity	Violent Felony	6047	14%
	Violent Misdemeanor	8901	20%
	Non-violent Felony	13,437	30%
	Non-violent Misdemeanor	15,925	36%
Prior Offenses	3+ Priors	2872	6%
	2 Priors	1821	4%
	1 Prior	4417	10%
	No Priors	35,200	79%
Pretrial Detention	Youth Detained Pretrial	14,273	32%

### Measures

#### Qualifying offense and court filings

This study uses the youth’s first offense in the five-year observation window from which to observe outcomes. This offense, designated as a qualifying offense (QO), was measured as the first court filing. A court filing indicated that a prosecutor judged the offense to meet the minimal standard of probable cause. Year in which the QO occurred was also included in the analysis.

#### Prior offenses

Prior offenses were defined as court filings that occurred before the QO as defined above. Further data were available on number of prior misdemeanor offenses and number of prior felony offenses. Number of prior offenses was strongly correlated with both prior misdemeanors (*r* = 0.87) and prior felonies (*r* = 0.74) and the use of prior offenses specified by offense type did not significantly change the accuracy of the model. Therefore, for analysis we generated discrete categories for number of priors (0 = no priors, 1 = one prior, 2 = two priors, and 3 = three or more priors), which allowed for assessment of the impact of increasing number of prior offenses while reducing skew.

#### Offense seriousness

Measures of offense severity of the QO available in the data included a continuous law severity scale (− 1 to 142) provided by the administrative data set, as well as whether the QO was a felony or a misdemeanor, and whether the QO was violent. Because the law severity scale was developed as a local administrative tool, we elected to categorize severity by misdemeanor, felonies, and violent offenses for improved generalizability and to limit redundancy. Furthermore, the law severity scale correlated well with felony offenses (*r* = 0.876), but not very well with violent offenses (*r* = 0.383), suggesting the impact of violent offenses was not captured well by the severity scale. Ultimately, a single severity variable was used spanning non-violent misdemeanors up to violent felonies (0 = non-violent misdemeanor, 1 = non-violent felony, 2 = violent misdemeanor, 3 = violent felony).

#### Demographics: age, gender, race

The data included the youth’s age at QO as well as age at the time of their first ever offense. As these two variables were highly correlated (*r* = 0.764), we selected the age at QO for the analytic models given that this was temporally related to the decision for pretrial detention. These data contained a gender variable that was predominantly limited to binary assignments of male or female with only 12 cases noted as “Other/Unspecified”.

Race and ethnicity groupings provided by the dataset included Caucasian, Black/African American, Asian, American Indian/Alaskan Native, Native Hawaiian/Pacific Islander, Hispanic, and Unknown/Other. Given the small sample size of Native Hawaiian/Pacific Islanders (*n* = 13), members of this category were placed into the Unknown/Other group for analysis. It should be noted that racial/ethnic identity for administrative records is intended to be solicited from individuals and that solicitation can occur multiple times throughout a youth’s process of system involvement: at the point of arrest by the arresting officer, at intake screening at the detention facility, through the juvenile prosecutor’s office, or other court processes. These processes likely introduce instances where race is assigned rather than self-identified, possible misassignment, or correction of prior error in some cases. Additionally, there was no further disaggregation of these categories, such as would be appropriate for the diversity of Asian individuals. These processes exemplify the above discussion of racialization as a verb and we must again highlight that these are socially constructed categories.

#### County

The county in which the QO occurred was available for each subject. Given the likelihood of variability in detention outcomes between counties, we considered county as a level 2 variable in our model. All other independent variables were considered level 1 variables.

#### Outcome variable: pretrial detention

Pretrial detention was indirectly measured as a detention stay occurring within 7 days of QO (0 = no pretrial detention, 1 = yes pretrial detention), presuming that youth detained immediately were detained in response to the court filing and not because of parole violations or sentencing following court hearings. The distribution of days between court filing and detention admission was extremely right skewed (median = 5 days, mean = 65 days, range = 0 to end of observation at 365 days). Over half of the sample was detained within 5 days (51%) and only an additional 800 cases (3%) were gained by extending the time to 10 days. Because of this, along with the relationship between detention soon after court filing, we counted any detention stay within 7 days of filing as an instance of pretrial detention.

### Analytic models

**Fig. 2:**
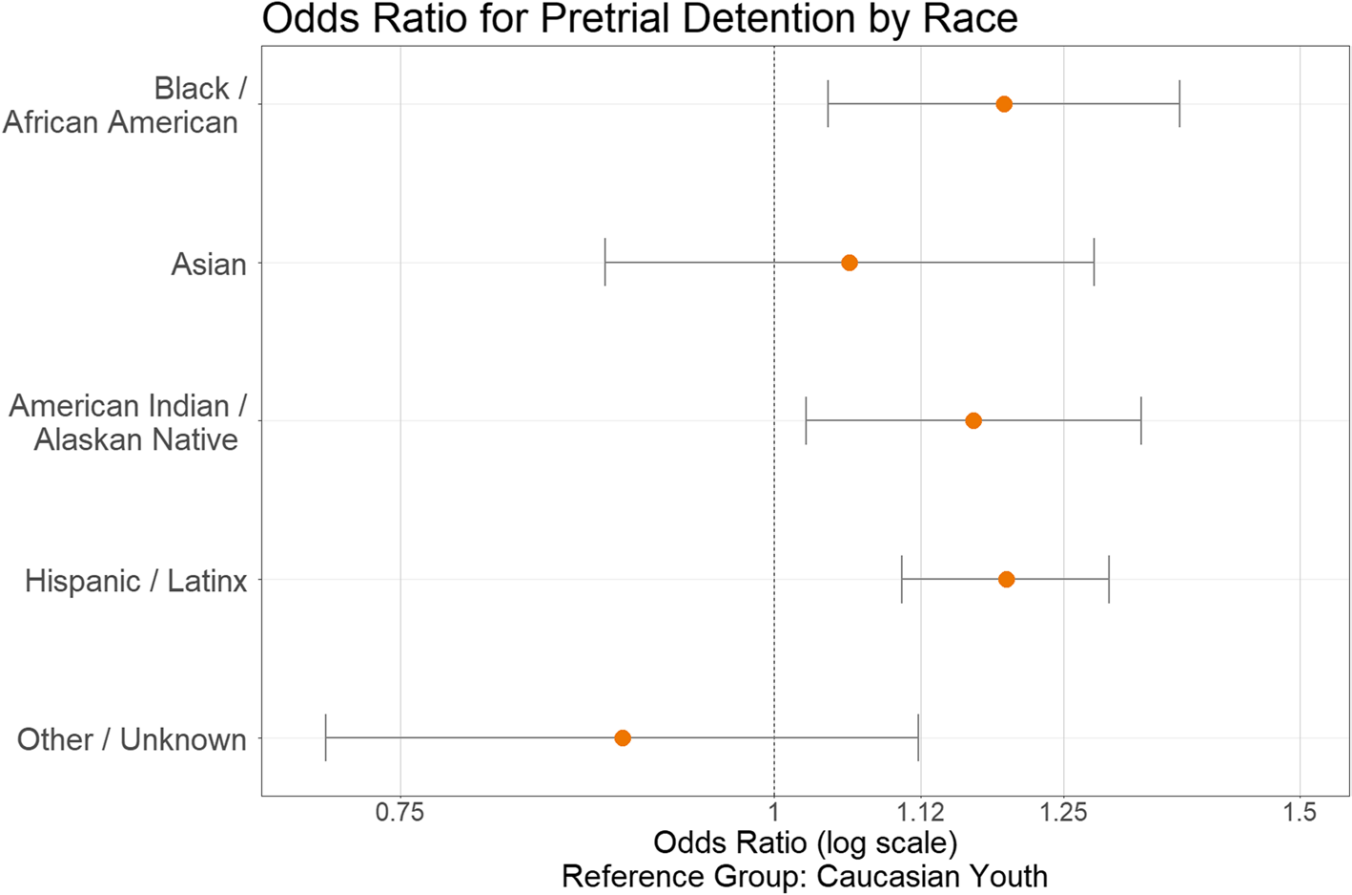
Odds ratio for pretrial detention by race

**Fig. 3:**
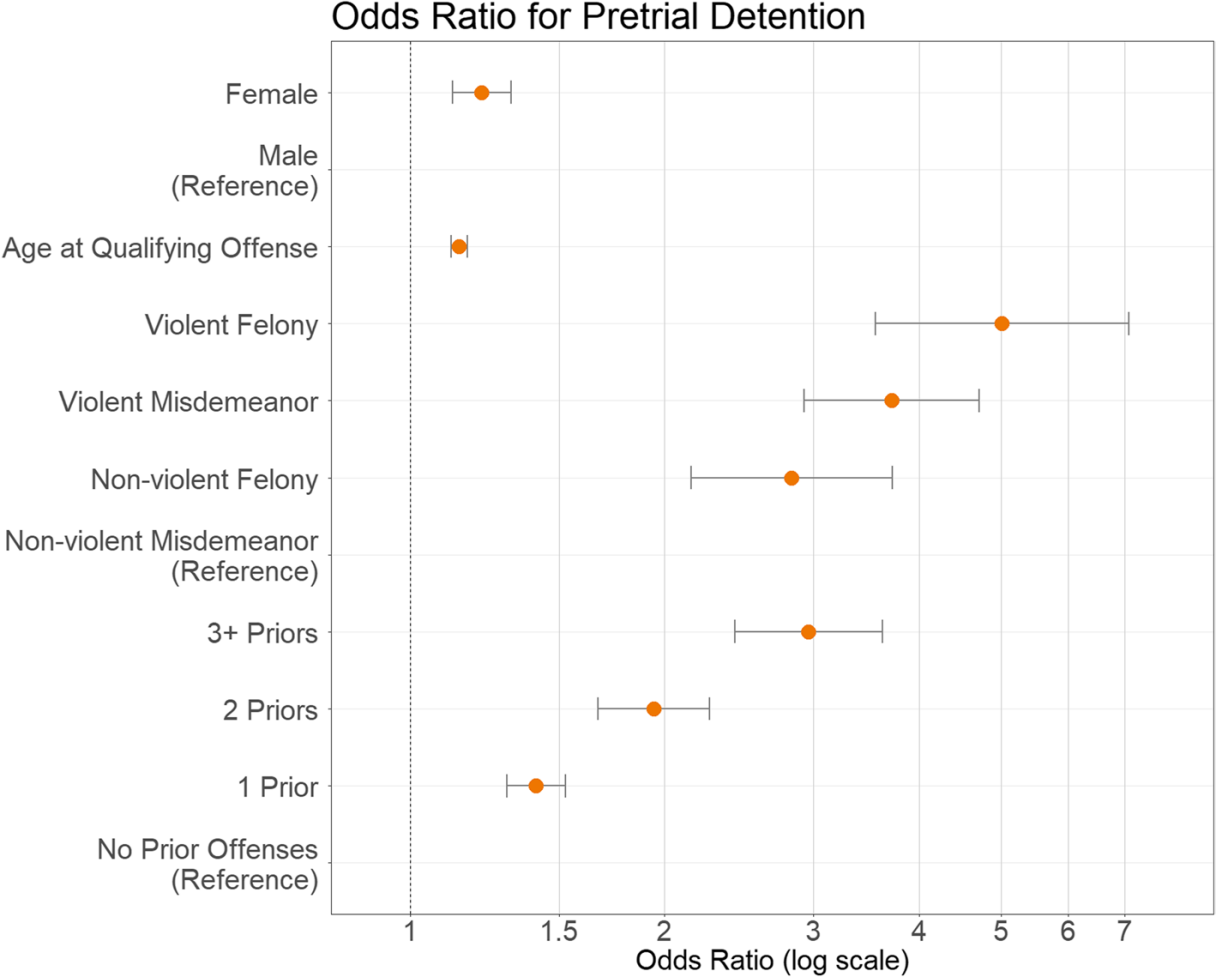
Odds ratio for other predictors

Analyses were performed in SPSS (Version 21). Because the outcome variable was dichotomous and cases were organized in a nested manner within counties, a generalized linear mixed model was used to estimate the effect of individual level characteristics while accounting for the random effect of differences at the county level. An intercept-only model was first estimated to determine if pretrial detention varied across counties. The random effects component of the intercept indicated significant variance by county (estimate = 0.25, *p* < 0.01) with an intraclass correlation coefficient of 0.20. The remaining individual level variables were included as predictors at level 1. Estimated regression coefficients, robust standard errors, and odds ratios calculated by exponentiating the estimated regression coefficients are presented below in Table 2. Figs. 2 and 3 presenting these results were created in R (Version 3.6.1).

## Results

Results revealed significant racial/ethnic disparities as shown in Fig. 2. Black/African American youth, American Indian/Alaskan Native youth, and Hispanic/Latinx youth were respectively 1.19 [95% CI: 1.04, 1.37], 1.17 [1.03, 1.33], and 1.20 [1.10, 1.30] times more likely than white youth to experience pretrial detention. There was no significant difference in odds of pretrial detention between white youth and Asian youth or youth categorized as Other/Unknown. Results for other predictors are displayed in Fig. 3. Gender disparity was present with female youth experiencing a 1.22 [1.12, 1.32] times higher likelihood of pretrial detention. Older youth were more likely to be detained pretrial with a log-odds of 0.13 [0.11, 0.16]. Qualifying offense severity was a significant predictor of pretrial detention. Compared to non-violent misdemeanors, non-violent felonies were 2.83 [2.15, 3.72] times as likely, violent misdemeanors were 3.71 [2.92, 4.71] times as likely and violent felonies were 5.01 [3.55, 7.08] times as likely to lead to pretrial detention. Each successive prior offense also increased the likelihood of detention. Youth with one prior offense had 1.41 [1.30, 1.53] times the likelihood of detention as those with no priors. For youth two priors there was 1.94 [1.67, 2.26] times the likelihood and youth with three or more priors had 2.96 [2.42, 3.62] times the likelihood of detention compared to youth without priors. There were no significant differences between years in which the QO occurred.

**Table 2:** Generalized linear mixed model estimates of pretrial detention

	*b* (SE)	OR (95% CI)
Intercept	−3.896 (0.294)***	0.020 (0.011–0.036)
Black / African American	0.177 (0.069)**	1.194 (1.043–1.367)
Asian	0.059 (0.096)	1.060 (0.878–1.280)
American Indian / Alaskan Native	0.154 (0.066)*	1.166 (1.025–1.327)
Hispanic / Latinx	0.179 (0.041)***	1.196 (1.104–1.295)
Other / Unknown	−0.117 (0.117)	0.890 (0.708–1.118)
White	(ref)	
Female	0.195 (0.041)***	1.216 (1.123–1.317)
Male	(ref)	
Age at Qualifying Offense	0.134 (0.011)***	1.143 (1.119–1.169)
Violent Felony	1.612 (0.176)***	5.013 (3.549–7.081)
Violent Misdemeanor	1.312 (0.122)***	3.712 (2.924–4.713)
Non-violent Felony	1.039 (0.140)***	2.827 (2.149–3.719)
Non-violent Misdemeanor	(ref)	
3+ Priors	1.085 (0.103)***	2.960 (2.421–3.620)
2 Priors	0.664 (0.078)***	1.942 (1.668–2.261)
1 Prior	0.344 (0.041)***	1.410 (1.301–1.528)
No Prior Offenses	(ref)	

Robust standard errors in parentheses. *OR =* odds ratio, derived from exponentiated b. (ref) = reference group. *p ≤ 0.05. **p ≤ 0.01. ***p ≤ 0.001

## Discussion

The primary goal of this study was to elucidate racial and ethnic disparities in the likelihood of pretrial detention. Although our analyses show that Black, Hispanic/Latinx, and American Indian/Alaskan Native youth are more likely to experience pretrial detention than white youth, likelihood of pretrial detention for Asian youth was not significantly different from white youth. This is consistent with social theory describing Asian Americans as the “Model Minority” who benefit from “positive” stereotypes and receive relative privilege in comparison to other communities of color (Kim, 1999). However, lack of disaggregated data for Asian Americans and Pacific Islanders is a frequent and significant limitation in epidemiologic studies that may be masking subgroup disparities (Jung et al., 2015; Ramakrishnan & Ahmad, 2014). Similarly, data on gender were limited to binary categories of assigned sex. As such we were not able to assess for disparities among gender non-conforming/LGBTQ+ youth—another overrepresented group in the juvenile carceral system (Wilson et al., 2017). Regarding gender, our study did find increased likelihood of pretrial detention for females compared to males. Overall, the female proportion of teen arrests grew from 20% to 30% between 1990 and 2010 (Sickmund et al., 2019). Our analyses adjusted for year of qualifying offense in an attempt to take into account such secular trends. However, the finding that females were more likely to be held in pretrial detention may be due to empirically observed gendered pathways into the justice system in which females are more likely to be arrested for family or peer conflict or running away, which can then lead to being charged with detainable offenses (Feld, 2009). Further considering intersectionality, while we did not test the interaction between race and gender, previous research demonstrates bias in decision-making for Black girls, in particular, leading to harsher sanctions than other demographic groups (Miller et al., 2021).

Another limitation is that the available data for analysis lacked additional youth-specific indicators such as school attendance, youth or parental mental health status, housing, and family structure and income. The ability to compare truly “similarly situated” youth would have enabled a stronger isolation of race as a predictor given that those with similar offenses or number of prior offenses may receive different judicial treatment because of different needs (Piquero, 2008). However, as discussed above in the introduction, other studies with these data available have demonstrated persistent racial disparity for youth in similar contexts. Likewise, even when administrative data is available on these constructs, it often reflects racial bias in categorization or ascertainment – for example, Black youth in many school districts are more likely to be categorized as truant than White youth even given the same number of total absences from school due to barriers and biases in how absences are judged excused vs. unexcused. As a result, adjusting for these constructs as measured in typical administrative data has the potential to unintentionally mask existing racial biases. Macro-level variables detailing differences between counties such as demographic make-up, economic indicators, and urban vs. non-urban settings which may have improved model fit and contributed to comparing similarly situated youth were lacking. Even so, our model controlled for county level variation broadly and the primary focus of the study was to focus on individual level variables as predictors. Lastly, our sample also did not include one of the largest (and most diverse) urban areas in the state which may have skewed representation toward rural counties.

Our study found that legal variables were stronger predictors of detention than race. Increasing severity of crime predicted detention, with higher odds for violent crimes compared to non-violent crimes and higher odds for felonies compared to misdemeanors. Each successive prior offense significantly increased the likelihood of detention, with youth with three or more priors being three times as likely to be detained than youth without prior offenses. Nevertheless, with these variables accounted for, significant racial/ethnic disparities are present. Moreover, our methods do not take into consideration other cumulative effects of race including increased policing, surveillance, and arrest rates for communities of color which directly impact number of priors—a significant predictor of pretrial detention (Davis et al., 2018; Pierson et al., 2020). While our study is somewhat limited in isolating racial bias within specific policies, practices or individuals embedded in the process of detaining youth, our findings show that the carceral institution (re) produces racial disparities for youth presenting with largely similar legal circumstances. What follows is a higher exposure to subsequent negative consequences.

We have seen that pretrial detention is predictive of worse outcomes at later stages of processing and is associated with recidivism (Feld, 1989; Leiber & Fox, 2005; Rodriguez, 2010; Walker & Herting, 2020). We have also seen youth exposed to direct harm in the detention setting and an independent association between incarceration during adolescence and poor health outcomes later in life (Barnert et al., 2017; Massoglia, 2008). It follows that the iatrogenic effects of detention are disproportionately imposed upon youth of color—particularly Black, Indigenous, and Hispanic/Latinx youth. Furthermore, disproportionality in incarceration has served as a covariate indicator of structural racism that predicts health outcomes (Lukachko et al., 2014; M. Wallace et al., 2017; M. E. Wallace et al., 2015). In this way, we can see how this carceral process operates as a mechanism of racialized social stratification as put forth by CRT. Remaining oriented toward impact and outcomes, the disparities demonstrated by our study present further evidence of institutional racism. That is, this institution through its policies and practices ultimately contributes to the ongoing construction of structural racism.

## Conclusion

After factoring in gender, age, crime severity, previous offenses, and variation between counties, our analyses show that Black, Hispanic/Latinx, and American Indian/Alaskan Native youth are more likely to experience pretrial detention than white youth. The likelihood of pretrial detention for Asian youth and for youth identified as “Other” or “Unknown” was not significantly different from white youth. As the iatrogenic harms of detention are disproportionately imposed upon youth of color—particularly Black, Indigenous, and Hispanic/Latinx youth—the disparities present in our study reveal further evidence of institutional racism. In this way, we can see how this carceral process operates as a mechanism of racialized social stratification as put forth by CRT. Considering implications for policy or further research, persistent disparity highlights an enduring need for building or strengthening diversion programs and alternatives to the carceral system, with emphasis on those that are culturally responsive.

Addressing disproportionate minority contact has been established as a core requirement of the Juvenile Justice and Delinquency Prevention Act since 1992 (Legislation, 2018). Despite this, our finding of disparate outcomes for youth of color in pretrial detention is consistent with research spanning four decades. This is emblematic of CRT’s tenets of both ordinariness and structural determinism. Considering implications for policy or further research, persistent disparity may highlight an enduring need for building or strengthening culturally responsive diversion programs that prioritize youth of color. To be clear, despite lack of significant evidence for disparity, Asian youth may also benefit from culturally responsive diversion. At least one meta-analysis has demonstrated that culturally adapted mental health interventions are significantly more effective (Griner & Smith, 2006). However, even in the presence of diversion programs, Black youth have been shown to be less likely to be selected for participation, which necessitates persistent vigilance in outcome monitoring (Leiber & Fox, 2005).

Troublingly, nearly 1 in 3 children in our study experienced incarceration before trial. Massoglia (2008) highlights that exposure to the penal system appears to produce significant health stratification for all individuals. Even with eliminated disparity, the proportion of youth who live through a detention episode is striking and speaks to the scarcity of available resources that prevent or provide alternatives to incarceration. While assessment and monitoring of disparities in pretrial detention are important, keeping any youth out of detention altogether is a preventative measure against poor health outcomes. Funding, developing, and evaluating alternatives such as diversion or transformative justice programs that do not rely upon the carceral system should be a priority for the benefit of all youth. Especially so, given that youth detention centers are sites of violence and harm and with convincing evidence suggesting that communities can reduce the use of detention without attendant increases in crime (Stahlkopf et al., 2010). Pending a commitment to build more of what Ruth Wilson Gilmore calls “life-affirming institutions,” detention will likely remain a default solution (Kaba & Nicholls, 2018).

## Data Availability

The data that support the findings of this study are available from the Washington State Administrative Office of the Courts (WA-AOC). but restrictions apply to the availability of these data, which were used under license for the current study, and so are not publicly available. Data are, however, available from the authors upon reasonable request and with permission of the WA-AOC.

## Footnotes

1 While typically called the juvenile justice system, the term juvenile carceral system has been intentionally used throughout this paper per recommendations from the Berkeley Underground Scholars Language Guide developed in partnership with formerly incarcerated people (Underground Scholars Language Guide, 2019)
